# Fish Oil Attenuates Omega-6 Polyunsaturated Fatty Acid-Induced Dysbiosis and Infectious Colitis but Impairs LPS Dephosphorylation Activity Causing Sepsis

**DOI:** 10.1371/journal.pone.0055468

**Published:** 2013-02-06

**Authors:** Sanjoy Ghosh, Daniella DeCoffe, Kirsty Brown, Ethendhar Rajendiran, Mehrbod Estaki, Chuanbin Dai, Ashley Yip, Deanna L. Gibson

**Affiliations:** Department of Biology, University of British Columbia Okanagan, Kelowna, British Columbia, Canada; National Institutes of Health, United States of America

## Abstract

Clinically, excessive ω-6 polyunsaturated fatty acid (PUFA) and inadequate ω-3 PUFA have been associated with enhanced risks for developing ulcerative colitis. In rodent models, ω-3 PUFAs have been shown to either attenuate or exacerbate colitis in different studies. We hypothesized that a high ω-6: ω-3 PUFA ratio would increase colitis susceptibility through the microbe-immunity nexus. To address this, we fed post-weaned mice diets rich in ω-6 PUFA (corn oil) and diets supplemented with ω-3 PUFA (corn oil+fish oil) for 5 weeks. We evaluated the intestinal microbiota, induced colitis with *Citrobacter rodentium* and followed disease progression. We found that ω-6 PUFA enriched the microbiota with Enterobacteriaceae, Segmented Filamentous Bacteria and *Clostridia* spp., all known to induce inflammation. During infection-induced colitis, ω-6 PUFA fed mice had exacerbated intestinal damage, immune cell infiltration, prostaglandin E2 expression and *C. rodentium* translocation across the intestinal mucosae. Addition of ω-3 PUFA on a high ω-6 PUFA diet, reversed inflammatory-inducing microbial blooms and enriched beneficial microbes like *Lactobacillus* and *Bifidobacteria*, reduced immune cell infiltration and impaired cytokine/chemokine induction during infection. While, ω-3 PUFA supplementation protected against severe colitis, these mice suffered greater mortality associated with sepsis-related serum factors such as LPS binding protein, IL-15 and TNF-α. These mice also demonstrated decreased expression of intestinal alkaline phosphatase and an inability to dephosphorylate LPS. Thus, the colonic microbiota is altered differentially through varying PUFA composition, conferring altered susceptibility to colitis. Overall, ω-6 PUFA enriches pro-inflammatory microbes and augments colitis; but prevents infection-induced systemic inflammation. In contrast, ω-3 PUFA supplementation reverses the effects of the ω-6 PUFA diet but impairs infection-induced responses resulting in sepsis. We conclude that as an anti-inflammatory agent, ω-3 PUFA supplementation during infection may prove detrimental when host inflammatory responses are critical for survival.

## Introduction

The effects of nutrition on health are extremely complex. In the gastrointestinal (GI) tract, dietary antigens interact with both the microbiota and the host mucosal surface which serves as a gatekeeper to systemic homeostasis. Certain dietary choices alter the ecology of the microbiota (known as dysbiosis; reviewed in [1]), and both dietary lipid intake and the intestinal microbiota have been identified as important factors contributing to the etiology of inflammatory bowel diseases (IBD). Dysbiosis has been observed in IBD patients, and the microbiota has been shown to induce or even prevent colitic disease in mouse models [2], [3]. We recently demonstrated that the intestinal microbiota protects against lethal infectious colitis by regulating protective inflammatory responses [2] and plays a major role in modulating pharmacological efficacy of drugs to attenuate colitis [4]. However, little is known about the effects of dietary lipids on inducing specific microbial changes that are either protective or detrimental in IBD. This is an important area of research since in recent years there have been profound changes in dietary fatty acid composition in parallel to the rapid rises in chronic inflammatory disorders including IBD.

In the past few decades, the lipid content of the “Western” diet has shifted from saturated fats to predominantly ‘cardioprotective’ ω-6 polyunsaturated fatty acids (PUFAs), of which the intake has risen by 54% over the last three decades in Canada [5]. Such benefits of ω-6 PUFAs are currently in question as new evidence reveals a detrimental effect of excess ω-6 PUFA intake in chronic metabolic disorders [6]. The specific impact of PUFAs on gut health are less clear, however consumption of high-fat diets [7] and specifically diets high in ω-6 PUFA [8] are risk factors for IBD in humans. Although linoleic acid (18∶2ω-6) is considered an essential nutrient, and required at 0.5–2% of total calories for proper development [9], North Americans currently consume between 5–10% dietary energy from ω-6 PUFA [10]. This is due to increased intake of ω-6 PUFA-rich oils such as sunflower, safflower and corn, as well as consumption of farm animals raised on oil seeds rich in ω-6 PUFA [11]. In parallel, the consumption of fish oils, rich in long chain ω-3 PUFAs like docosahexaenoic acid (DHA) and eicosapentaenoic acid (EPA), which are responsible for numerous anti-inflammatory effects, represent only 0.15% of dietary energy in the North American diet [12]. As a result of this imbalance, there has been interest in supplementing our diets with sources of ω-3 PUFAs such as fish oil pills which have been suggested to prevent symptoms of IBD [13]. However, the effects of ω-3 PUFA supplementation on gut health are conflicting with some studies showing they are beneficial [8], [14], [15], while others demonstrate they exacerbate colitis [16], [17]. It is not known how the combination of ω-6 and ω-3 PUFAs affect gut microbes and IBD susceptibility.

In this study, we hypothesized that excess dietary ω-6 PUFA would increase colitis susceptibility through the microbe-immunity nexus. C57BL/6 mice were fed high-fat diets (20% w/w) that varied in PUFA composition: corn oil (high ω-6 PUFA diet); corn oil+fish oil (ω-3 PUFA supplemented diet) and a low-fat diet composed of ω-6 PUFA (5% w/w corn oil) as a control. We evaluated the intestinal microbiota, induced colitis with the enteric bacterial pathogen *Citrobacter rodentium* and followed disease progression. ω-6 PUFA enriched pro-inflammatory gut microbiota and exacerbated intestinal damage and inflammation during infection. Supplementing the ω-6 PUFA diets with ω-3 PUFA reversed these pro-inflammatory blooms and instead enriched anti-inflammatory microbes corresponding with less intestinal damage. However, ω-3 PUFA supplementation also reduced the presence of intestinal alkaline phosphatase (IAP) expressing cells resulting in the inability to detoxify LPS. The fish oil supplemented diet resulted in increased mortality during infection due to sepsis evident by the presence of serum LPS binding protein (LBP), IL-15 and TNF-α. We conclude that excess dietary ω-6 PUFA enriches pro-inflammatory microbes and augments colitis. In contrast, fish oil supplementation reverses the effects of an ω-6 PUFA rich diet but may prove detrimental to the host during infection when inflammatory responses are critical for survival.

## Results

### ω-6 PUFA Rich Diets Cause Dysbiosis While Fish Oil Supplementation can Reverse these Effects

**Figure 1:**
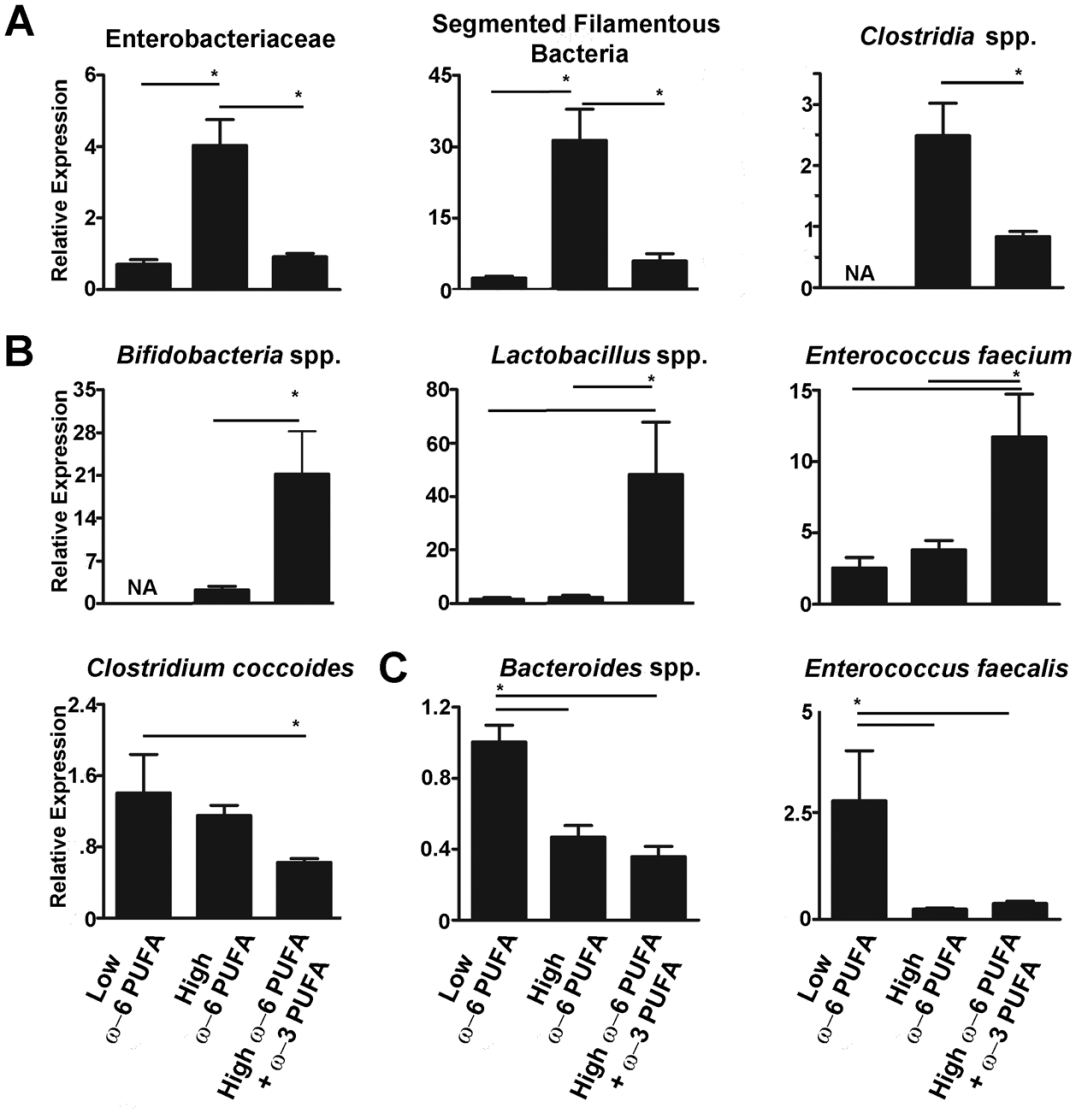
ω-6 PUFA rich diets induce dysbiosis and ω-3 PUFA supplementation reverses these blooms while enriching beneficial microbes. A) ω-6 PUFA rich diets promote a microbiota enriched with Enterobacteriacea, Segmented Filamentous Bacteria (SFB) and microbes from the *Clostridia coccoides* group and ω-3 supplementation reverses this and B) enriches *Bifidobacteria* spp., *Lactobacillus* spp. and *Enterococcus faecium*. C) High-fat diet groups resulted in decreased *Bacteroides* spp. and *Enterococcus faecalis*. Expression is relative to the low ω-6 PUFA group. (*, *P*<0.05).

To examine the effects of PUFAs on the intestinal microbial ecology, we used qPCR and primers specific to the 16S rRNA sequences from clinically important microbes which are altered in IBD patients or experimental models of intestinal inflammation [4]. ω-6 PUFA rich diets enriched the microbiota with Enterobacteriaceae (Figure 1A), which are associated with IBD [18], [19], [20], [21], and Segmented Filamentous Bacteria (SFB; Figure 1A), previously shown to induce responses that drive experimental colitis [22]. In contrast, ω-3 PUFA supplementation prevented these specific enrichments. While both high-fat diets increased *Clostridia* spp. compared to the low ω-6 PUFA control, the ω-3 PUFA supplemented group had reduced abundance compared to the ω-6 PUFA-rich diet (Figure 1A). The ω-3 PUFA supplemented group had fewer microbes from the *Clostridium coccoides* group (Figure 1B) which are opportunistic pathogens associated with IBD [23]. Besides the lack of potential pathobionts with ω-3 PUFA supplementation, this group also had enriched populations of the beneficial microbes *Lactobacillus* spp. and *Bifidobacteria* spp., (Figure 1B) known to be anti-inflammatory during murine colitis [24], [25]. ω-3 PUFA supplementation also induced *Enterococcus faecium* (Figure 1B) which has reported probiotic properties [26]. In general, both high-fat diets reduced *Bacteroides* spp. (Figure 1C) a trend associated with obesity [27], and correspondingly both groups of mice were similarly obese (data not shown). Both high-fat diets reduced the levels of *Enterococcus faecalis* compared to the low ω-6 PUFA control (Figure 1C), also reported to have probiotic properties [26]. Overall, while ω-6 PUFA rich diets induced the growth of microbes known to induce pro-inflammatory responses, ω-3 PUFA supplementation reversed this and in parallel enriched beneficial bacteria.

### ω-6 PUFA Rich Diets Cause Severe Colonic Damage and while ω-3 PUFA Prevents this Damage it Leads to Increased Mortality during *C. rodentium* Infection

**Figure 2:**
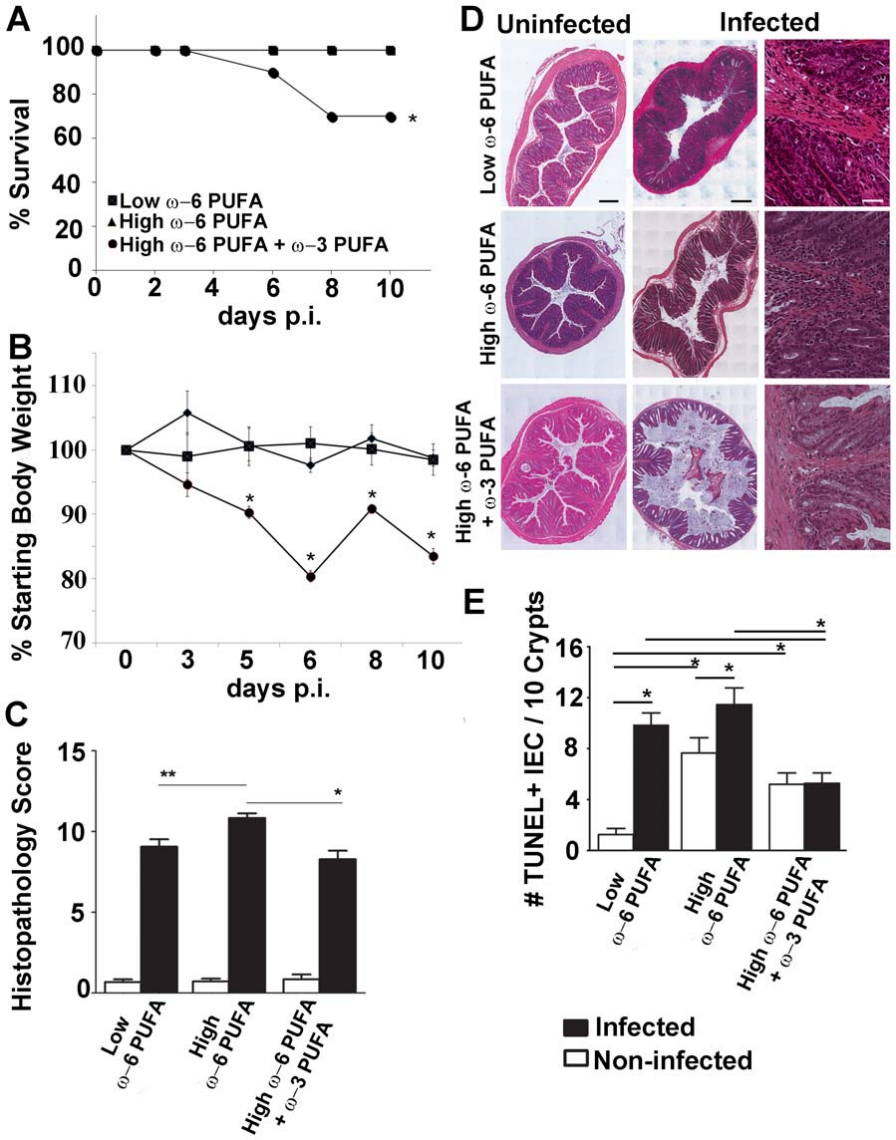
While ω-3 PUFA supplementation prevents ω-6 PUFA induced histopathologic severity, these mice suffered increased mortality and morbidity during *C. rodentium* infection. A) C57BL/6 mice fed ω-6 PUFA rich diets supplemented with ω-3 PUFA suffered increased mortality during infection with *C. rodentium*. 30% of mice fed ω-3 PUFA required euthanization by 6–8 days p.i., in contrast to the low or high-fat ω-6 PUFA groups. B) While both low or high-fat ω-6 PUFA diets result in similar weight changes during infection with *C. rodentium*, the ω-3 PUFA supplemented group suffered significantly increased cachexia throughout days 5–10 p.i. C) Mice fed ω-6 PUFA rich diets had the highest induction of histopathologic severity during *C. rodentium*-induced colitis while ω-3 PUFA supplementation reduced this. D) Representative colon sections from diet groups were taken at 100× magnification and stitched together using Metamorph software (black scale bar = 46.5 µm) or 200× magnification (white scale bar = 20.7 µm). E) Colonic tissue sections were stained for TUNEL-positive cells. Colons from mice fed low and high-fat ω-6 PUFA diets showed increased cell death compared to mice fed diets supplemented with ω-3 PUFA. (*, *P*<0.05).

To determine the effects of PUFA diets on intestinal inflammation, we used *C. rodentium,* an enteric bacterial pathogen that induces acute colitis, to examine intestinal responses in mice fed high-fat PUFA diets. The normally resistant C57BL/6 mice fed ω-3 PUFA supplemented diets suffered increased mortality during infection where 30% of the mice had to be sacrificed by day 8 post-infection (p.i.; Figure 2A). While both low and high ω-6 PUFA diets resulted in similar weight changes during infection, the ω-3 PUFA supplemented group suffered the greatest weight loss throughout days 5–10 p.i. (Figure 2B) despite similar pre-infection body weights and caloric intake in mice fed both high-fat diets (data not shown). Yet during infection, the ω-3 PUFA fed mice displayed similar histopathological severity (based on mucodepletion, hyperplasia, immune cell infiltration, edema and epithelial integrity) as to the low ω-6 PUFA control (Figure 2C & D). In contrast, ω-6 PUFA-rich fed mice displayed the most severe histopathology (Figure 2C–D). We examined intestinal epithelial cell death to determine the effect of PUFA diets on intestinal homeostasis since this correlates with intestinal damage [28], [29]. Both high-fat diets induced cell death prior to acute colitis revealing that epithelial cell homeostasis was disrupted by high-fat alone (Figure 2E). Following infection, mice fed ω-6 PUFA rich diets had increased colonic cell death (Figure 2E) whereas ω-3 PUFA supplementation attenuated this response indicating that a high ω-6: ω-3 PUFA ratio induces exacerbated colonic damage.

### ω-6 PUFA Rich Diets Increase Intestinal Inflammatory Cell Infiltration while ω-3 PUFA Supplementation Impairs Infection-induced Immune Responses

**Figure 3:**
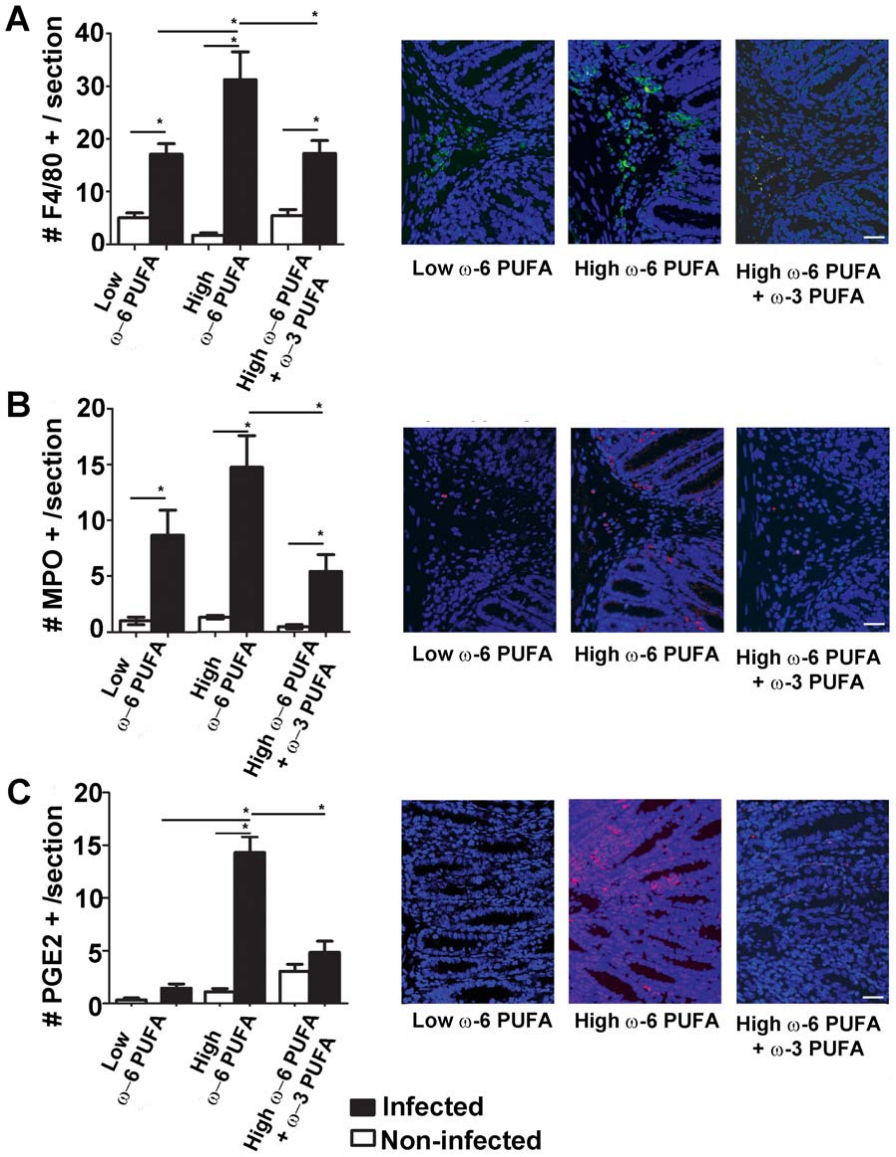
ω-3 PUFA supplementation to ω-6 PUFA rich diets impairs intestinal inflammatory cell infiltration during *C. rodentium-*induced colitis. While ω-6 PUFA rich diets induced the infiltration of macrophages, neutrophils and PGE2 inflammatory cells, ω-3 PUFA supplementation prevented the enhanced infiltration during infection. Colon sections were stained for the presence of submucosal A) F4/80+ macrophages B) MPO+ neutrophils and C) PGE2+ cells and quantified. Representative immunofluorescence images are shown at 200× magnification. (scale bar = 13.6 µm; *, *P*<0.05).

ω-6 PUFA rich diets enriched pro-inflammatory-inducing microbes and worsened colitis, while ω-3 PUFA supplementation reversed these effects and enriched beneficial microbes. To further investigate the inflammatory status of these mice, we examined intestinal immune responses in tissues from mice collected both before and during *C. rodentium* infection. We examined colonic neutrophil and macrophage cell infiltration (Figure 3A–B) and found that ω-6 PUFA rich diets increased infiltration of F4/80+ macrophages and MPO+ neutrophils compared to the low ω-6 PUFA group during infection. In contrast, ω-3 PUFA supplementation restored immune cell infiltration similar to the low ω-6 PUFA control. We also examined the levels of PGE2, a key inflammatory marker in the gut, and found that mice fed ω-6 PUFA rich diets had the highest infection-induced levels of colonic PGE2. In contrast, ω-3 PUFA supplementation did not induce PGE2+ cell infiltration during infection (Figure 3C) suggesting that these mice were impaired in mounting an inflammatory response to infection.

**Figure 4:**
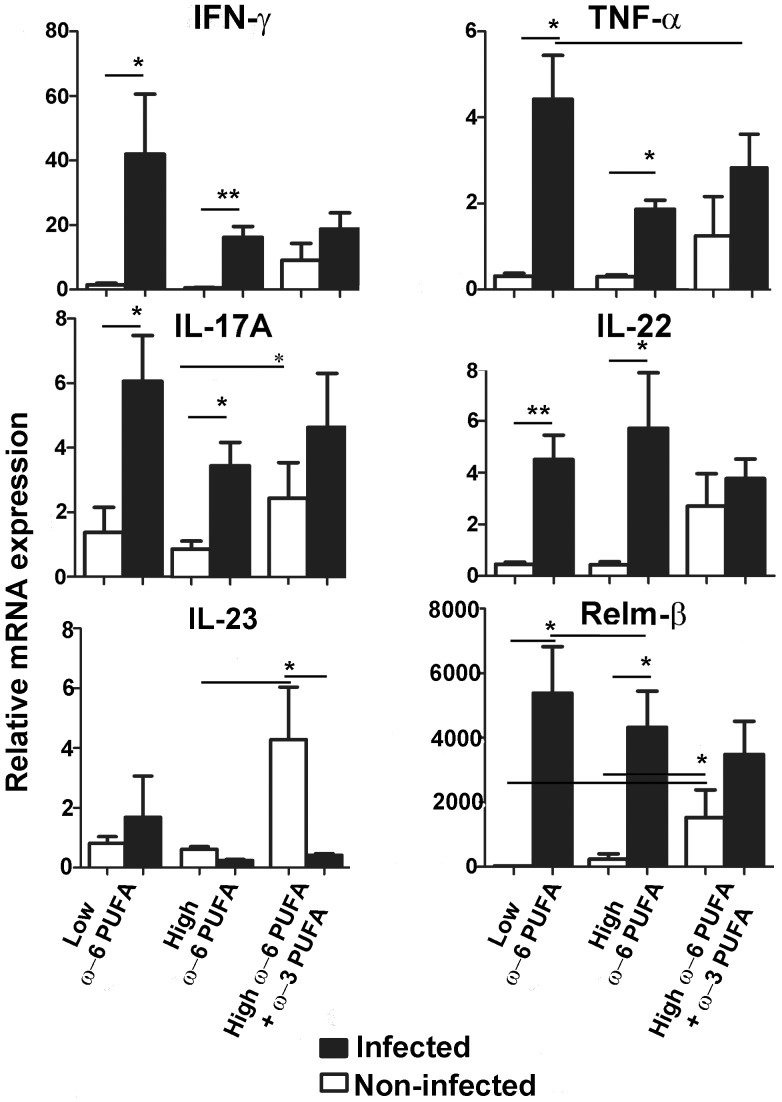
ω-3 PUFA supplementation to ω-6 PUFA rich diets impairs infection-induced cytokine and chemokine responses. qPCR analysis of colonic tissues revealed that both high and low ω-6 PUFA diets were relatively similar where infection induced cytokine and the chemokine Relm-β responses. However, the ω-3 PUFA supplemented diet had impaired IFN-γ, TNF-α, IL-17A, IL-22 and IL-23, as well as the Relm-β responses evident by the lack of their induction during infection. (*, *P*<0.05; **, *P*<0.005).

To examine which cytokines were modulated as a result of PUFA feeding, we examined responses important during *C. rodentium*-induced colitis [28], [29]. Neither of the high-fat diets exacerbated cytokine responses during infection compared to the low fat control (Figure 4). In general, the ω-6 PUFA rich fed mice were able to mount an inflammatory response evident by the induction of cytokine expression post-infection. In contrast, the ω-3 PUFA supplemented group was unable to induce such responses during infection evident by the lack of induction of IFN-γ, TNF-α, IL-17A, IL-22 and IL-23, as well as the chemokine Relm-β compared to pre-infection expression (Figure 4). This data reveals that ω-3 PUFA supplementation impairs infection-induced cytokine responses even though ω-3 PUFA supplemented mice had increased IL-23 and Relm-β expression under uninfected conditions (Figure 4). Finally, we examined adiponectin since its impaired expression in mice fed ω-3 PUFA was shown to be responsible for increased colitis and mortality when exposed to dextran sodium sulfate (DSS) [17]. We found that infection reduced adiponectin expression similarly in both high-fat diets (data not shown) and concluded that the lack of adiponectin was not responsible for the increased mortality observed in the ω-3 PUFA supplemented group.

### ω-6 PUFA Rich Diets Resulted in Increased Translocation of γ-Proteobacteria

**Figure 5:**
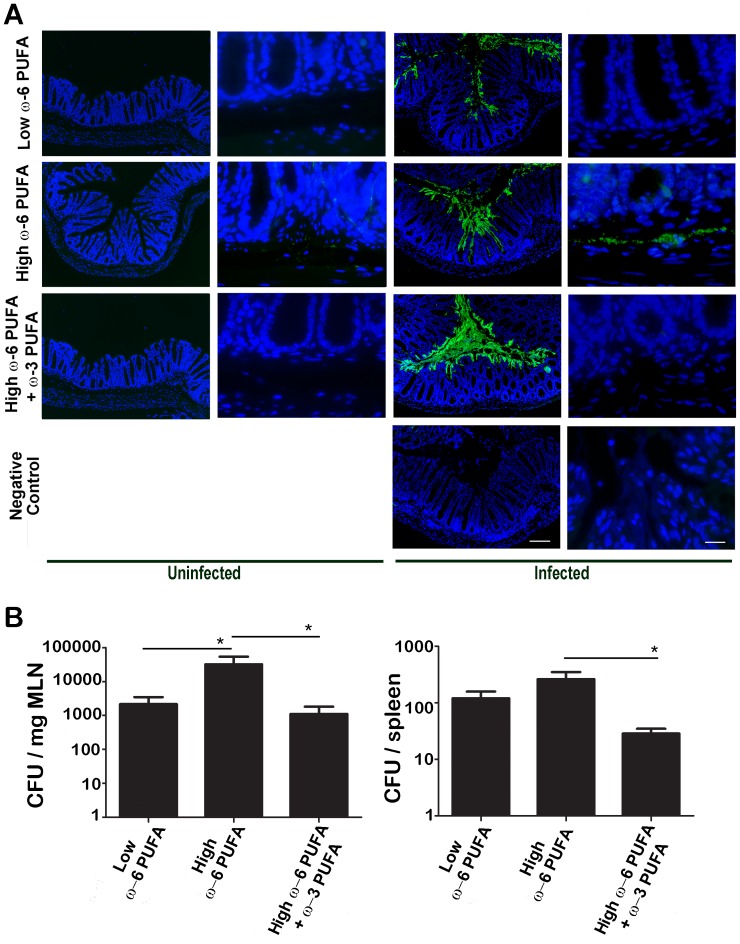
ω-6 PUFA rich diets resulted in increased translocation of microbes from Enterobacteriacea across the intestinal mucosae. A) Colonic tissue sections were hybridized with a γ-Proteobacteria probe (green) and the nuclei stained with DAPI (blue) to examine the locations of Enterobacteriaceae. These Gram-negative microbes were found in the submucosae (600× magnification scale bar = 14.2 µm) prior to infection in the colons of mice fed ω-6 PUFA rich diets. During infection with *C. rodentium*, a member of Enterobacteriaceae, the pathogen was found deep into the crypts (100× magnification, scale bar = 85.4 µm; upper panel) and in the submucosae (600× magnification scale bar = 14.2 µm) in the colons of mice fed ω-6 PUFA rich diets. B) Colony forming units (CFU) recovered from the spleen and mesenteric lymph nodes (MLN) were highest from mice fed ω-6 PUFA rich diets after 10 days of *C. rodentium* infection. CFU were enumerated from tissues removed from mice fed various diets and were homogenized followed by plating in serial dilutions on Lb agar. (*, *P*<0.05).

Since we found increased levels of Enterobacteriaceae in mice fed ω-6 PUFA rich diets and these mice showed the most severe colitis, we examined the location of these microbes on colonic tissue sections hybridized with a γ-Proteobacteria probe (Figure 5A) to determine if these microbes were interacting with immune cells. While few microbes were present prior to infection they were found in the submucosal region of the mice fed high ω-6 PUFA diets (600× magnification) while none were present in the mice fed diets supplemented with ω-3 PUFA. Because *C. rodentium* is a member of γ-Proteobacteria, we could also use this probe to examine the localization of the pathogen during infection. We found the microbes deeply invading the crypts (100× magnification), and were observed in the submucosal region (600× magnification) of the colons from mice fed ω-6 PUFA**-**rich diets. In contrast, the mice fed ω-3 PUFA supplemented diets were able to contain the pathogen at the top of the crypts and bacteria were not observed in the submucosal region. This suggested that the pathogen was more systemic in the mice fed ω-6 PUFA rich diets but that fish oil supplementation prevents this bacterial translocation. To confirm this, we examined the spleen and mesenteric lymph nodes (MLN) for *C. rodentium* colony forming units (CFU). We found an increase in CFU in the tissues from mice fed ω-6 PUFA rich diets, which was reduced following ω-3 PUFA supplementation (Figure 5B).

### ω-3 PUFA Supplementation Induces Sepsis in *C. rodentium* Infected Mice

**Figure 6:**
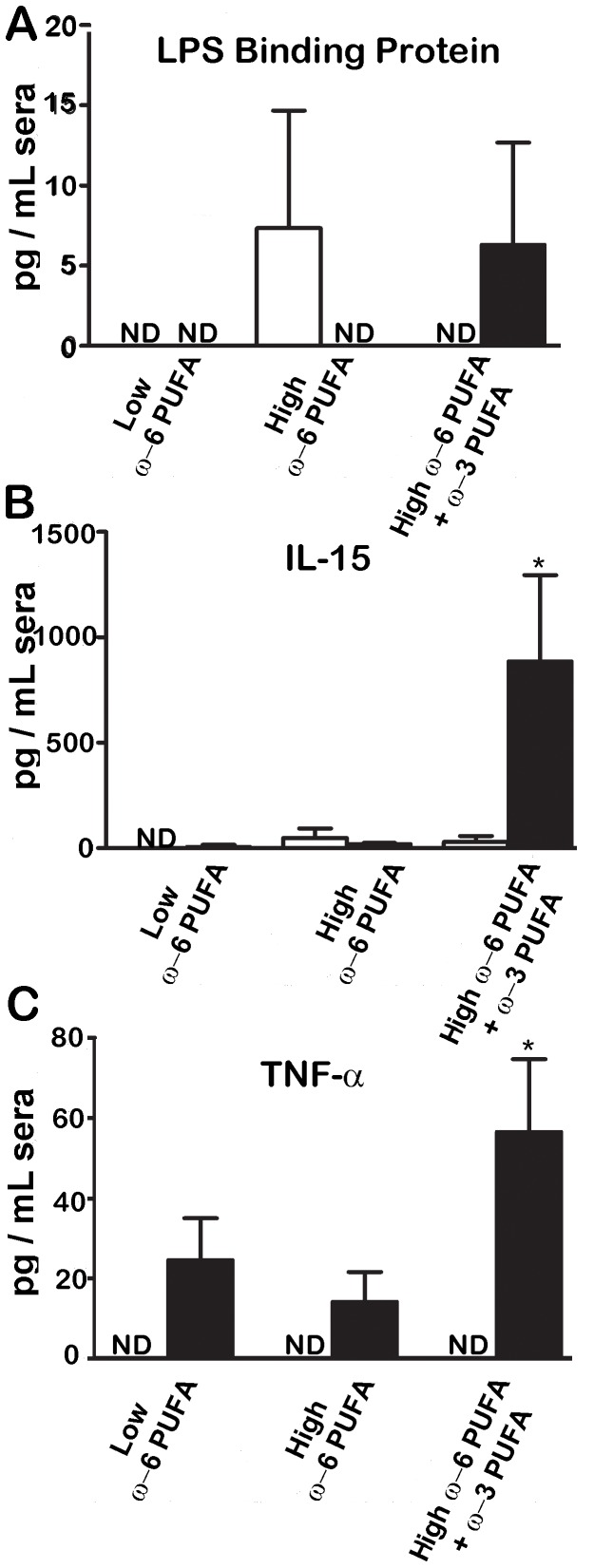
While ω-6 PUFA rich diets resulted in the induction of LPS binding protein prior to infection, it was the ω-3 PUFA supplemented diet that induced infection-induced septic responses. LPS binding protein, TNF-α and IL-15 were measured in sera from in mice that had been fed the various diets both before and after infection with *C. rodentium*. (*, *P*<0.05).

To determine if the translocation of the pathogen had an effect on systemic inflammation, we examined sera for the presence of LPS binding protein (LBP), a clinical marker of sepsis [30]. In support of the observed translocation of *C. rodentium* across the mucosal surface in the high ω-6 PUFA fed mice, we found LBP in the sera of these mice prior to infection (Figure 6A). In contrast, there was no induction of LBP in mice fed ω-3 PUFA supplements prior to infection but during infection LBP levels increased. Because ω-3 PUFA supplemented mice suffered increased mortality, we also examined serum IL-15, a cytokine shown to induce sepsis [31], as well as TNF-α (Figure 6B & C). Despite there being lower levels of pathogen in the spleen and MLN in the ω-3 PUFA supplemented group, these mice had the greatest induction of serum IL-15 and TNF-α. These results suggested that while ω-6 PUFA rich diets resulted in increased bacterial translocation under uninfected conditions, they were able to avoid infection-induced sepsis. In contrast, ω-3 PUFA supplementation inhibited bacterial translocation in the absence of infectious insult, but these mice suffered sepsis following *C. rodentium* infection.

### Intestinal Alkaline Phosphatase Expression and LPS Dephosphorylation Activity was Impaired in Mice fed ω-3 PUFA Supplemented Diets during Infection

**Figure 7:**
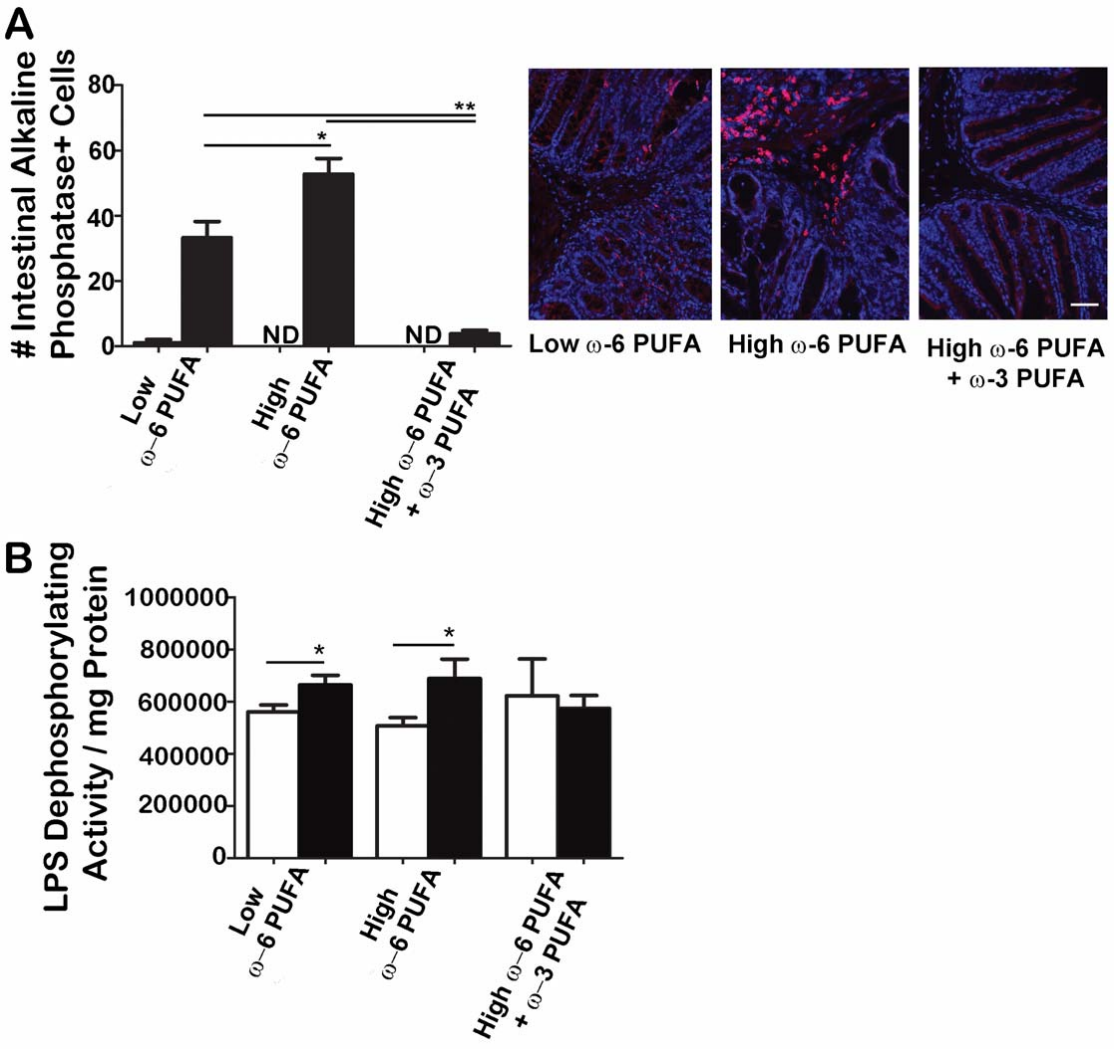
ω-3 PUFA supplementation to ω-6 PUFA rich diets resulted in impaired infection-induced intestinal alkaline phosphatase activity. A) IAP+ cells were highest in ω-6 PUFA rich diet groups during infection and ω-3 PUFA supplementation impaired this response. Colon sections were stained for the presence of IAP+ cells and quantified. Representative immunofluorescence images are shown at 200× magnification (scale bar = 13.6 µm). B) While both the low and high ω-6 PUFA rich diet groups showed an induction of LPS-dephosphorylating activity during infection induced colitis, theω-3 PUFA supplementation fed mice were unable to dephosphorylate LPS in response to infection. Colonic tissues were homogenized, supernatant collected and LPS incubated with each diet group for 2 hours. A colorimetric malachite green solution was used to measure absorbance at 620 nm and the LPS-dephosphorylating activity/mg of protein was determined for each diet group. (*, *P*<0.05; **, *P*<0.005).

To determine what factors could be causing sepsis despite decreased systemic pathogen translocation in mice fed ω-3 PUFA supplemented diet, we examined the expression of intestinal alkaline phosphatase (IAP), a key endogenous mucosal defense factor inducible by microbiota [32]. IAP dephosphorylates LPS during infection preventing sepsis [33], [34]. We found the greatest number of IAP+ cells infiltrating in the colonic submucosae from mice fed ω-6 PUFA rich diets (Figure 7A). In contrast, mice fed diets supplemented with ω-3 PUFA had fewer IAP+ cells. Since IAP detoxifies LPS through dephosphorylation [34], we examined the *ex vivo* LPS dephosphorylation activity of colons from mice fed PUFA diets similar to Goldberg *et al* (2008) [34]. While both low and high ω-6 PUFA rich diets induced LPS-dephosphorylating activity during *C. rodentium* infection, the ω-3 PUFA supplemented diet resulted in an impaired ability to dephosphorylate LPS during infection (Figure 7B). Overall, these results show that ω-3 PUFA supplementation reduces pathogen translocation but also impairs IAP expression and the ability to dephosphorylate LPS during infection. As a result, any pathogen that escapes across the barrier in ω-3 PUFA supplemented mice may be more toxic leading to increased systemic inflammation associated with the increased mortality during *C. rodentium* infection.

## Discussion

An increase in IBD incidence has been associated with increased dietary intake of ω-6 PUFA in humans [8], while IBD patients remain in remission longer when dietary intake of ω-3 PUFA increases [14], . In mice, an ω-6 PUFA rich diet promotes immune responses to bacteria [36], and enhances mucosal damage during trinitrobenzene sulfonic acid-induced colitis [37]. In contrast, diets low in ω-6 PUFA and high in ω-3 PUFA decrease spontaneous colitis in IL-10 knockout mice [38]. Furthermore, endogenously produced ω-3 PUFAs in transgenic fat-1 mice, reduces COX-2 and PGE2 expression protecting against DSS-colitis [39]. Despite such positive evidence, other studies show that diets rich in ω-3 PUFA exacerbate murine colitis through impaired expression of intestinal adiponectin [17]. Similarly, the offspring of rats fed fish oil perinatally have exacerbated colitis when challenged with DSS in adulthood [16]. Thus, while the overabundance of dietary ω-6 PUFA may cause excessive gut inflammation, the effects of fish oil supplementation on colitis are unclear. In this study, ω-6 PUFA rich diets increase intestinal damage and inflammation during infection-induced colitis. Supplementation with fish oil, which contains ω-3 PUFA, reversed these effects; however several colonic responses during infection were severely impaired including immune cell infiltration, cytokine/chemokine induction and LPS dephosphorylation as a result of reduced IAP+ infiltrating cells. Consequently, mice fed diets supplemented with ω-3 PUFA suffered from increased infection-induced mortality associated with sepsis related serum LBP, IL-15 and TNF-α.

We found that diets rich in ω-6 PUFA produced microbiota with increased numbers of potential pathobionts including bacteria from the family Enterobacteriaceae. This is similar to IBD patients [18], [19], [20], [21], and mice undergoing DSS colitis [40], supporting the hypothesis that ω-6 PUFA diets increase intestinal microbes which drive colitis. We also found these pro-inflammatory microbes were present in the colonic submucosa, and while we did not observe overt colitis prior to infection, we did find presence of serum LBP. This suggests that ω-6 PUFA rich diets prior to infectious insult, promote inappropriate inflammation. Following *C. rodentium* infection, these mice suffered severe colitis. Similarly, ω-6 PUFA diets increase the susceptibility for TNBS-induced enteritis [37]. We also found high levels of IAP+ cells infiltrating the colonic tissues of infected mice that had been fed ω-6 PUFA rich diets. IAP has been associated with an influx of inflammatory cells during DSS-colitis [33].

Remarkably, diets supplemented with ω-3 PUFA prevented the bloom of Enterobacteriaceae, as well as the translocation of bacteria into the submucosal region and instead promoted the enrichment of *Lactobacillus* spp. and *Bifidobacteria* spp., both of which are associated with milder DSS and *C. rodentium* induced colitis [40], [41]. ω-3 PUFA supplementation prevented pathogen translocation, which may be due to the increase in *Lactobacillus* spp. and *Bifidobacteria* spp., since both are known to increase barrier function [42], [43]. Akin to earlier reports demonstrating decreased levels of mucosal PGE2 following ω-3 PUFA administration in colitis [39], ω-3 PUFA fed mice displayed reduced immune cell infiltration and a lack of infection-induced up-regulation of PGE2 and cytokine responses. While *C. rodentium*-induced inflammation promotes colonic damage in C57BL/6 mice [2], these responses are also important for *C. rodentium* clearance [28], [29]. Our results suggest that ω-3 PUFA supplementation to an ω-6 PUFA rich diet results in a milder colitis but impairs infection-induced inflammatory responses important for preventing systemic *C. rodentium* infection. Similarly, another study demonstrated that mice fed high levels of ω-3 PUFAs had impaired immune function and could not produce a response against *Helicobacter hepaticus*-induced infection [44]. Consumption of ω-3 PUFA rich diets may have anti-inflammatory properties however this may prevent the body from mounting appropriate immune responses critical for host defense.

In following with this, the mice fed ω-3 PUFA supplemented high-fat diets suffered increased mortality during *C. rodentium* infection. While the lack of colonic inflammatory responses likely played a role, a contributing factor was the inability to detoxify LPS through the lack of dephosphorylation activity in response to *C. rodentium* infection. In addition, there was a lack of IAP+ submucosal cells infiltrating into the colons of these mice. IAP prevents sepsis [32] by dephosphorylating LPS [34]. While the pathogen did not translocate across the intestinal barrier at an increased level in the mice fed diets supplemented with ω-3 PUFA, *C. rodentium* itself induces barrier dysfunction [45]. Thus, some pathogen became systemic and this resulted in sepsis. In support of this, mice fed fish oil supplemented diets had elevated serum-associated LBP, a clinical biomarker of sepsis associated with mortality [46]. Additionally, in these mice infection induced serum IL-15 and TNF-α expression. IL-15 induces sepsis [47] as determined with IL-15 knockout mice which avoid sepsis through the lack of protease activation [31].

In conclusion, we found that excessive consumption of dietary ω-6 PUFA increases colitis through the microbe-immunity nexus. We also found that clinically recommended amounts of ω-3 PUFA supplementation reverses the effects of ω-6 PUFA rich diets. While this may be beneficial in the context of chronic colitis, it is detrimental in the context of an acute gut infection. Together, these data reveal that the colonic microbiota can be altered through exogenous dietary PUFA intake and varying ratios of dietary ω-6: ω-3 PUFA can alter susceptibility to infection-induced colitis by modulating inflammatory and cytoprotective responses.

## Materials and Methods

### Mice

C57BL/6 mice (Jackson Laboratories; Bar Harbor, Maine) were maintained at the Center for Disease Modeling at the University of British Columbia (UBC), Vancouver, BC. They were bred in house and caged in a temperature-controlled (22±2°C) room with a 12 hr light/dark cycle and fed irradiated food and tap water under specific pathogen-free conditions.

### Ethics

The protocols used were approved by the UBC’s Animal Care Committee and in direct accordance with guidelines drafted by the Canadian Council on the Use of Laboratory Animals.

### Post-natal Diet Model

Female offspring were weaned at 4 weeks of age onto isocaloric, isonitrogenous high-fat diets with 40% energy or chow diets with 9% energy from fats. High-fat diets contained 20% w/w of various oils prepared by blending dietary oils (Table 1) to a rodent basal diet mix from Harlan Teklad (catalog# TD.88232; Table 2) and then fed diets for 5 weeks. The diet oil components include: corn oil (high ω-6 PUFA diet), and corn oil+fish oil (ω-3 PUFA supplemented diet). For ω-3 PUFA supplementation, we followed the guidelines set by the American Heart Association recommending 0.5–1.8 g of long chain ω-3 PUFAs per day to offset high ω-6 PUFA diets. As EPA+DHA are 34% of fish oil by weight, 0.5 g of EPA+ DHA is available in 1.5 g of fish oil. As 18 g of ω-6 PUFA is present in 30 g of corn oil; we added 1% fish oil (w/w) with 19% (w/w) corn oil. Low ω-6 PUFA-fed animals (5% w/w corn oil; Harlan Teklad, cat. #8640) were also included in the study as a control. Food and water was provided *ad libitum*.

**Table 1:** Major fatty acid compositions of dietary oil used in preparing high-fat diets.

FA	Corn Oil (%)	Soybean Oil (%)	Fish Oil* (%)
Saturated FA	10.8	13.8	31.4
Linoleic Acid	61.7	55.4	1.6
Arachidonic Acid	0	0	2.5
Alpha Linolenic acid	1.2	7.7	0.3
Oleic Acid	26.1	22.4	22.1
DHA +EPA	0	0	34

**Table 2:** Composition of high-fat diets.

Formula per Kg of all HF diets in g		
	High ω-6 PUFA	High ω-6+ ω-3 PUFA
Casein	240	240
DL-Methionine	3.6	3.6
Corn Starch	150	150
Sucrose	298.8	298.8
Cellulose	50	50
Calcium carbonate	3.6	3.6
Mineral Mix^1^	42	42
Vitamin Mix^2^	12	12
Soybean Oil^3^	20	20
**Corn Oil**	180	170
**Fish Oil**	0	10
Total	1000	1000

1 Mineral mix (mg/g): di calcium phosphate 500, magnesium oxide 24; potassium citrate 220, potassium sulfate 52; sodium chloride 74, chromium KSO_4_ 12H_2_0 0.55; cupric carbonate 0.3, potassium iodate 0.01; ferric citrate 6, manganous carbonate 3.5, sodium selenite 0.01, zinc carbonate 1.6; sucrose 118.03.

2 Vitamin Mix (mg/g): vitamin A 0.8; vitamin D_3_ 1; vitamin E 10; menadione sodium bisulfite 0.08; nicotinic acid 3; calcium pantothenate 1.6; pyridoxine HCl 0.7; riboflavin 0.6; thiamin 0.6; sucrose 978.42.

3 Added to meet essential fatty acid requirements for all groups.

### *Citrobacter Rodentium* Infection and Morbidity/Mortality Measurement

*C. rodentium* DBS100 were cultured overnight as previously reported [29]. Mice were infected via oral gavage and monitored throughout the 10 day infection for mortality/morbidity and were euthanized if they lost more than 20% of their initial body weight or showed other signs of extreme distress. Survival data are presented as percentage of the initial mice still surviving at each time point and body weight data are presented as percentage of the initial body weight.

### Tissue Collection

Mice were euthanized by cervical dislocation, the distal colon removed (stool was removed) and immersed in RNA-later® (Qiagen). Portions of the colon were either stored at −20°C for real time qPCR analysis, homogenized and stored at −80°C for bacterial DNA extraction or immersed in 10% neutral buffered formalin (Fisher) for histological analyses and immunofluorescence.

### Histopathological Scoring, Immunofluorescence and TUNEL Staining

Colonic histopathology was assessed using a scoring system previously described [2]. The average scores accumulated from colonic tissues were quantified by two blinded observers. Images were taken at 100× magnification and stitched together using MetaMorph® software for Olympus (http://support.meta.moleculardevices.com/docs/mm%20bag.pdf). For immunofluorescence and TUNEL staining, paraffin-embedded tissue sections were deparaffinized and antigen retrieval of rehydrated tissues was performed using a 1 mg/mL trypsin (Sigma) followed by incubation with either: rabbit polyclonal antibody-1 raised against myeloperoxidase (Thermo Scientific) to examine neutrophils; rat monoclonal antibody raised against F4/80 (CedarLane) to examine macrophages; rabbit polyclonal antibody raised against prostaglandin (PGE)2 (Abcam); and rabbit polyclonal antibody raised against IAP (Abcam) followed by either goat anti-rabbit IgG AlexaFluor-conjugated 594-red antibody (Invitrogen); goat anti-rabbit IgG 488-conjugated antibody (Rockland) or goat anti-rat IgG DiLyteFluor 488-labelled antibody (Ana Spec Inc). Tissue sections were mounted using fluoroshield with DAPI (Sigma) and viewed on an Olympus IX81 fluorescent microscope. Negative controls using secondary rat or rabbit antibodies only were examined and found negative**.** For inflammatory cell counts, positive cells were quantified by two blinded observers under fluorescence from a stitched image using MetaMorph® software. Cell death was measured using a FragEL DNA Fragmentation Detection Kit (Calbiochem) as per manufacturer’s instructions. The mean number of TUNEL-positive cells for each group was the total number of TUNEL-positive cells counted in 10 longitudinally sectioned crypts, imaged and quantified as above.

### mRNA Analysis

Total RNA was purified using Qiagen RNEasy kits (Qiagen) according to the manufacturer’s instructions. cDNA was synthesized with iScript cDNA Synthesis Kit (Bio-Rad). Quantitative PCR reactions were performed as described previously [4] using Bio-Rad CFX Manager 2.0 and Sso Fast Eva Green Supermix (Bio-Rad). All primers were synthesized by Integrated DNA Technology (IDT), Canada. Primer efficiencies were verified according to the minimum information for publication of quantitative real-time PCR experiments (MIQE) guidelines. Primers used were: 18S rRNA (F; CGGCTACCACATCCAAGGAA, R; GCTGGAATTACCGCGGCT), Relm-β (F; GCTCTTCCCTTTCCTTCTCCAA, R; AACACAGTGTAGGCTTCATGCTGTA), IL-23 (F; TGGCATCGAGAAACTGTGAGA, R; TCAGTTCGTATTGGTAGTCCTGTTA), TNF-α, IFN-γ, and IL-22 primers previously described [2], [48]. Expression of 18S rRNA was used as a reference for gene expression analysis and quantification of gene expression was carried out using CFX manager software version 1.6.541.1028 (Bio-Rad).

### Bacterial Analysis

To examine the mucosal associated intestinal microbiota, colon pieces were washed, homogenized and bacterial genomic DNA was extracted using a DNA stool minikit (Qiagen) according to the manufacturer’s instructions and quantified as above using 50 ng/µl of bacterial DNA. Primers are described previously [4]. Relative values for bacterial groups were normalized to total bacteria present amplified using a universal Eubacterial probe. Fluorescent *in situ* hybridization was performed using a FITC-labeled γ-Proteobacteria probe as previously described 4. Sections were imaged as described above. For systemic pathogen counts, the spleen and mesenteric lymph nodes were homogenized in PBS, serially diluted and plated on McConkey agar plates which were incubated for 24–48 hr at 37°C.

### Serum Cytokine Quantification

Blood was collected from mice via cardiac puncture, spun at 1600 g for 5 min at 4°C, to separate the serum. The serum was then analyzed for TNF-α using a commercially available ELISA kit (Biolegend) according to the manufacturer. A standard curve was established between 0 and 1,000 pg/ml and all samples were assayed in duplicates. LPS binding protein and IL-15 was measured from sera serviced by Evetechnologies (evetechnologies.com; Calgary, Canada).

### LPS Dephosphorylation Assay

To measure LPS- dephosphorylating activity, a protocol was produced from the work described by Goldberg *et al*. (2008) [34]. Colon tissues were homogenized in 500µL of homogenization buffer, centrifuged to remove insoluble material at 11,000 rpm for 3 min. To determine the protein concentrations a Bradford assay (Bio-Rad) was performed on the colon samples according to the manufacturer. A standard curve was created using stock solution of 1 mg/mL BSA (Sigma) in triplicates with multiple concentrations. 40 µL of a 5 mg/mL solution of *Escherichia coli* 055:B5 LPS (Sigma L2880) was then added to 15 µL lysate and left for 2 hours at room temperature. 40 µL of a solution composed of 0.01% malachite green (Sigma), 16% sulfuric acid (Fisher), 1.5% ammonium molybdate (Sigma) and 0.18% Tween-20 (Sigma) similar to Baykov *et* *al.* (1988) [49] was incubated with the lysate for 10 min. LPS- dephosphorylating activity was determined from colorimetric measurements taken at an absorbance of 620 nm.

### Statistical Analysis

The results are expressed as the mean +/− standard error of the mean where for uninfected mice: n = 12 for the high ω-6 PUFA group, n = 13 for the low ω-6 PUFA and high ω-6 PUFA+fish oil supplemented groups and for infected mice: n = 10 for the low and high ω-6 PUFA groups and n = 8 for the high ω-6 PUFA+fish oil supplemented groups). Significance for survival data was determined using the log-rank test. For qPCR data, minitab was used to test for normalcy and then one-way ANOVA with Tukey post hoc test was performed for parametric data and Krustal-Wallis with Dunns post-hoc test for non-parametric data. For weight loss, histological scoring, TUNEL+ cells, bacterial and host cell numbers, serum analysis and LPS- dephosphorylating assays data were analyzed via one-way ANOVA with Tukey post-hoc tests. All analyses were performed using GraphPad Prism 5 where *P*<0.05 was considered significant.
